# Methyl 4′-(4-fluoro­phen­yl)-1′-methyl-3′-nitro­methyl-2-oxospiro­[indoline-3,2′-pyrrolidine]-3′-carboxyl­ate

**DOI:** 10.1107/S1600536813001578

**Published:** 2013-01-23

**Authors:** B. K. Revathi, S. Sathya, G. Usha, G. Murugan, M. Bakthadoss

**Affiliations:** a Post Graduate and Research Department of Physics, Queen Mary’s College, Chennai-4, Tamilnadu, India; b Department of Organic Chemistry, University of Madras, Maraimalai Campus, Chennai-25, Tamilnadu, India

## Abstract

In the title compound, C_21_H_20_FN_3_O_5_, the the pyrrolidine ring makes dihedral angles of 84.91 (6) and 62.38 (7)° with the oxindole unit and the fluoro­phenyl ring, respectively. The pyrrolidine ring assumes an envelope conformation with the spiro C atom as the flap. The crystal packing features weak N—H⋯N and C—H⋯O hydrogen bonds.

## Related literature

For background to pyrrolidine derivatives, see: Raj *et al.* (2003 ▶); Cordell (1981 ▶); Usha *et al.* (2005 ▶). 
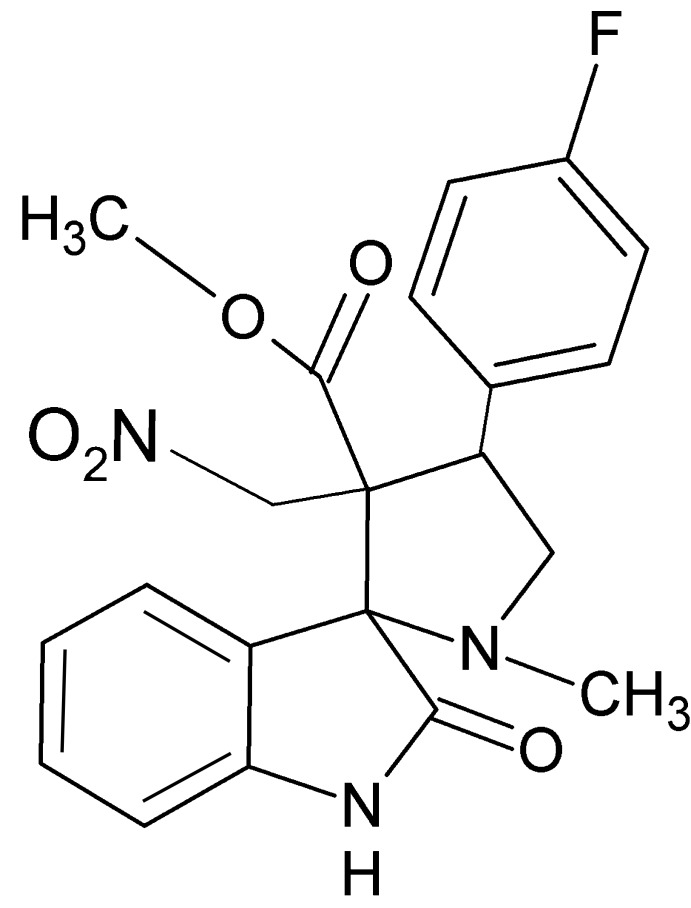


## Experimental

### 

#### Crystal data

C_21_H_20_FN_3_O_5_
*M* *_r_* = 413.40
Orthorhombic, 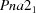

*a* = 8.0265 (5) Å
*b* = 25.7011 (15) Å
*c* = 9.7763 (6) Å
*V* = 2016.8 (2) Å^3^
*Z* = 4
Mo *K*α radiation
μ = 0.10 mm^−1^
*T* = 293 K
0.20 × 0.18 × 0.18 mm

#### Data collection

Bruker Kappa APEXII CCD diffractometer
Absorption correction: multi-scan (*SADABS*; Bruker, 2004 ▶) *T*_min_ = 0.979, *T*_max_ = 0.981
22124 measured reflections
2551 independent reflections
2355 reflections with *I* > 2σ(*I*)
*R*_int_ = 0.022

#### Refinement

*R*[*F* ^2^ > 2σ(*F* ^2^)] = 0.047
*wR*(*F* ^2^) = 0.132
*S* = 1.13
2551 reflections
271 parameters
1 restraint
H-atom parameters constrained
Δρ_max_ = 0.30 e Å^−3^
Δρ_min_ = −0.16 e Å^−3^

### 

Data collection: *APEX2* (Bruker, 2004 ▶); cell refinement: *SAINT* (Bruker, 2004 ▶); data reduction: *SAINT* and *XPREP* (Bruker, 2004 ▶); program(s) used to solve structure: *SHELXS97* (Sheldrick, 2008 ▶); program(s) used to refine structure: *SHELXL97* (Sheldrick, 2008 ▶); molecular graphics: *ORTEP-3 for Windows* (Farrugia, 2012 ▶); software used to prepare material for publication: *SHELXL97* and *PLATON* (Spek, 2009 ▶).

## Supplementary Material

Crystal structure: contains datablock(s) I, global. DOI: 10.1107/S1600536813001578/bh2472sup1.cif

Structure factors: contains datablock(s) I. DOI: 10.1107/S1600536813001578/bh2472Isup2.hkl

Supplementary material file. DOI: 10.1107/S1600536813001578/bh2472Isup3.cml

Additional supplementary materials:  crystallographic information; 3D view; checkCIF report

## Figures and Tables

**Table 1:** Hydrogen-bond geometry (Å, °)

*D*—H⋯*A*	*D*—H	H⋯*A*	*D*⋯*A*	*D*—H⋯*A*
N2—H2*A*⋯N1^i^	0.86	2.34	3.127 (2)	152
C4—H4⋯O1^i^	0.93	2.42	3.223 (2)	145
C9—H9*B*⋯O1^ii^	0.97	2.49	3.314 (2)	142
C9—H9*A*⋯O4^iii^	0.97	2.51	3.443 (3)	160

Symmetry codes: (i) 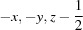; (ii) 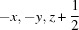; (iii) 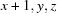.

**Fig. 1.**
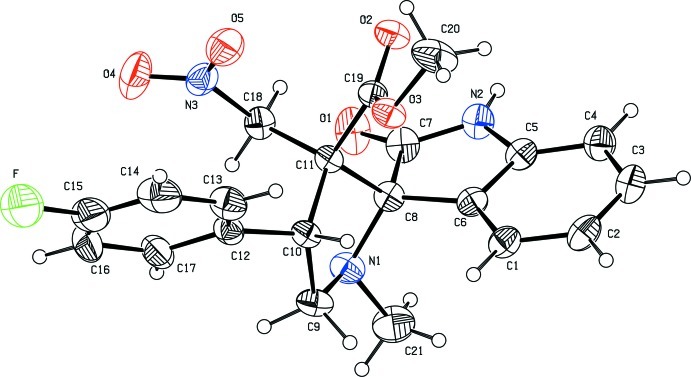
The molecular structure of the title compound, with displacement ellipsoids drawn at the 30% probability level.

**Fig. 2.**
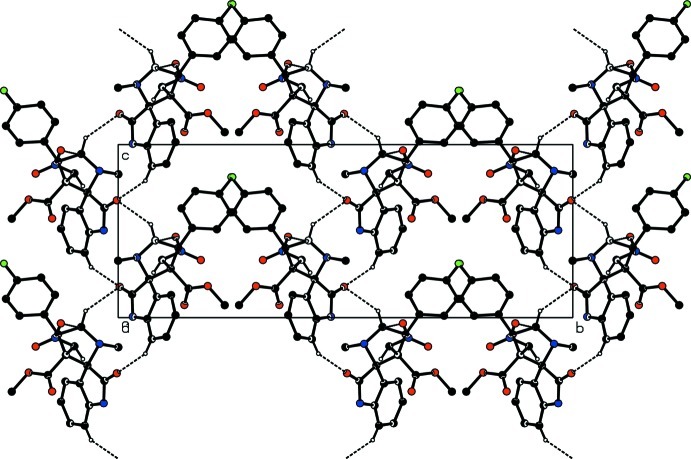
The packing of the molecules in the crystal structure. The dashed lines indicate the hydrogen bonds.

## supplementary crystallographic information

### Comment

Pyrrolidine ring system is a frequently encountered structural motif in many biologically relevant alkaloids (Cordell, 1981). Substituted pyrrolidines possess anti-microbial and anti-fungal activity against various pathogens (Raj *et al.*, 2003). In the title compound (Fig. 1), the N—C and C—C bond lengths are slightly longer than the normal values, but are comparable with those in other reported structures (*e.g.* Usha *et al.*, 2005). This may be due to the spiro-atom character and the steric forces of bulky substituents in the pyrrolidine moiety. Bond length C7—O1, 1.226 (3) Å, indicates a keto group character. The dihedral angle [84.91 (6)°] between oxindole moiety and pyrrolidine ring shows that they are almost orthogonal to each other. The sum of the angles around N2 atom, 360.0°, and N3 atom, 359.9°, is an indication of their *sp*^2^ hybridization. The O atoms of the nitro group have somewhat higher displacement parameters than the other atoms, indicating greater thermal motion for these atoms. The pyrrolidine ring adopts envelope conformation, with C8 as the flap.

The crystal packing is stabilized by weak N—H···N and C—H···O hydrogen bonds. The symmetry-related molecules linked through alternate N—H···N and C—H···O type hydrogen bonds are forming loops. These units running along [001] form molecular chains, which are connected by C—H···O hydrogen bonds, resulting in molecular layers (Fig. 2).

### Experimental

A mixture of (*E*)-methyl 3-(4-fluorophenyl)-2-(nitromethyl)acrylate (1 mmol, 0.24 g), isatin (1 mmol, 0.15 g) and sarcosine (1 mmol, 0.09 g) in acetonitrile (6 ml) was refluxed for 14 h. After completion of the reaction, as indicated by TLC, the reaction mixture was concentrated, and the resulting crude diluted with water (10 ml), and extracted with ethyl acetate (3 × 10 ml). The combined organic layers were washed with brine (2 × 10 ml) and dried over anhydrous Na_2_SO_4_. The organic layer thus obtained was concentrated under reduced pressure, and the residue was purified by column chromatography on silica gel (Acme 100–200 mesh), using ethyl acetate:hexanes (3:7), to afford the title compound as a colourless solid, in 68% yield.

### Refinement

All H atoms were positioned geometrically and treated as riding on their parent atoms, with C—H distances in the range 0.93–0.98 Å and N2—H2A distance of 0.86 Å. Displacement parameters for H atoms were calculated as and *U*_iso_(H) = 1.2*U*_eq_(N, C), except for the methyl groups, for which *U*_iso_(H) = 1.5*U*_eq_(C).

### Crystal data

**Table:**

C_21_H_20_FN_3_O_5_	*F*(000) = 864
*M**_r_* = 413.40	*D*_x_ = 1.362 Mg m^−^^3^
Orthorhombic, *P**n**a*2_1_	Mo *K*α radiation, λ = 0.71073 Å
Hall symbol: P 2c -2n	θ = 2.2–28.0°
*a* = 8.0265 (5) Å	µ = 0.10 mm^−^^1^
*b* = 25.7011 (15) Å	*T* = 293 K
*c* = 9.7763 (6) Å	Block, colourless
*V* = 2016.8 (2) Å^3^	0.20 × 0.18 × 0.18 mm
*Z* = 4	

### Data collection

**Table:**

Bruker Kappa APEXII CCD diffractometer	2551 independent reflections
Radiation source: fine-focus sealed tube	2355 reflections with *I* > 2σ(*I*)
Graphite monochromator	*R*_int_ = 0.022
ω and φ scan	θ_max_ = 28.0°, θ_min_ = 2.2°
Absorption correction: multi-scan (*SADABS*; Bruker, 2004)	*h* = −10→10
*T*_min_ = 0.979, *T*_max_ = 0.981	*k* = −33→33
22124 measured reflections	*l* = −12→12

### Refinement

**Table:**

Refinement on *F*^2^	Primary atom site location: structure-invariant direct methods
Least-squares matrix: full	Secondary atom site location: difference Fourier map
*R*[*F*^2^ > 2σ(*F*^2^)] = 0.047	Hydrogen site location: inferred from neighbouring sites
*wR*(*F*^2^) = 0.132	H-atom parameters constrained
*S* = 1.13	*w* = 1/[σ^2^(*F*_o_^2^) + (0.1*P*)^2^] where *P* = (*F*_o_^2^ + 2*F*_c_^2^)/3
2551 reflections	(Δ/σ)_max_ < 0.001
271 parameters	Δρ_max_ = 0.30 e Å^−^^3^
1 restraint	Δρ_min_ = −0.16 e Å^−^^3^
0 constraints	

### Fractional atomic coordinates and isotropic or equivalent isotropic displacement parameters (Å^2^)

**Table:**

	*x*	*y*	*z*	*U*_iso_*/*U*_eq_	
C1	0.3956 (3)	0.11630 (9)	0.1303 (3)	0.0495 (5)	
H1	0.4261	0.1306	0.2140	0.059*	
C2	0.4898 (3)	0.12602 (10)	0.0138 (3)	0.0565 (6)	
H2	0.5850	0.1465	0.0206	0.068*	
C3	0.4449 (3)	0.10596 (11)	−0.1112 (3)	0.0587 (6)	
H3	0.5085	0.1137	−0.1880	0.070*	
C4	0.3039 (3)	0.07382 (10)	−0.1246 (3)	0.0547 (5)	
H4	0.2729	0.0599	−0.2086	0.066*	
C5	0.2141 (3)	0.06398 (8)	−0.0077 (3)	0.0451 (5)	
C6	0.2544 (3)	0.08481 (7)	0.1197 (2)	0.0427 (4)	
C7	0.0156 (3)	0.03050 (8)	0.1341 (3)	0.0493 (5)	
C8	0.1232 (3)	0.06782 (7)	0.2227 (2)	0.0411 (4)	
C9	0.2291 (3)	0.08136 (9)	0.4461 (3)	0.0514 (5)	
H9A	0.3491	0.0856	0.4421	0.062*	
H9B	0.1982	0.0717	0.5386	0.062*	
C10	0.1406 (2)	0.13220 (7)	0.4033 (2)	0.0390 (4)	
H10	0.2258	0.1548	0.3629	0.047*	
C11	0.0209 (2)	0.11542 (7)	0.2839 (2)	0.0365 (4)	
C12	0.0607 (3)	0.16266 (9)	0.5181 (2)	0.0456 (5)	
C13	0.0601 (4)	0.21706 (11)	0.5106 (3)	0.0588 (6)	
H13	0.1131	0.2333	0.4375	0.071*	
C14	−0.0166 (5)	0.24707 (14)	0.6083 (4)	0.0829 (11)	
H14	−0.0177	0.2832	0.6014	0.099*	
C15	−0.0913 (5)	0.2224 (2)	0.7158 (4)	0.0902 (13)	
C16	−0.0913 (5)	0.1694 (2)	0.7311 (3)	0.0904 (13)	
H16	−0.1425	0.1539	0.8060	0.109*	
C17	−0.0125 (4)	0.13928 (14)	0.6316 (3)	0.0628 (7)	
H17	−0.0088	0.1033	0.6412	0.075*	
C18	−0.1491 (3)	0.09642 (8)	0.3340 (2)	0.0466 (5)	
H18A	−0.2032	0.0778	0.2601	0.056*	
H18B	−0.1318	0.0720	0.4082	0.056*	
C19	−0.0041 (3)	0.15462 (7)	0.1679 (2)	0.0390 (4)	
C20	0.0779 (4)	0.23421 (12)	0.0716 (4)	0.0769 (10)	
H20A	0.1486	0.2634	0.0914	0.115*	
H20B	−0.0350	0.2459	0.0619	0.115*	
H20C	0.1137	0.2180	−0.0119	0.115*	
C21	0.2968 (4)	−0.00037 (11)	0.3289 (4)	0.0769 (9)	
H21A	0.3231	−0.0156	0.4159	0.115*	
H21B	0.3961	0.0138	0.2889	0.115*	
H21C	0.2515	−0.0265	0.2694	0.115*	
N1	0.1739 (3)	0.04125 (7)	0.3481 (2)	0.0503 (4)	
N2	0.0717 (2)	0.03194 (7)	0.0047 (2)	0.0506 (5)	
H2A	0.0266	0.0154	−0.0622	0.061*	
N3	−0.2642 (2)	0.13883 (9)	0.3830 (2)	0.0558 (5)	
O1	−0.0977 (3)	0.00319 (8)	0.1779 (2)	0.0701 (6)	
O2	−0.0938 (2)	0.14544 (7)	0.0731 (2)	0.0534 (4)	
O3	0.0879 (2)	0.19697 (6)	0.18238 (18)	0.0467 (4)	
O4	−0.3674 (3)	0.12634 (12)	0.4665 (3)	0.0891 (8)	
O5	−0.2541 (3)	0.18170 (9)	0.3327 (3)	0.0725 (6)	
F	−0.1702 (4)	0.25155 (16)	0.8126 (3)	0.1407 (14)	

### Atomic displacement parameters (Å^2^)

**Table:**

	*U*^11^	*U*^22^	*U*^33^	*U*^12^	*U*^13^	*U*^23^
C1	0.0430 (10)	0.0469 (11)	0.0586 (13)	−0.0016 (9)	−0.0007 (10)	−0.0078 (10)
C2	0.0408 (10)	0.0505 (12)	0.0781 (17)	−0.0041 (9)	0.0071 (11)	−0.0067 (12)
C3	0.0542 (12)	0.0576 (13)	0.0644 (15)	−0.0041 (10)	0.0159 (12)	−0.0052 (12)
C4	0.0578 (12)	0.0523 (12)	0.0539 (12)	−0.0018 (10)	0.0035 (11)	−0.0060 (10)
C5	0.0449 (10)	0.0352 (9)	0.0553 (12)	0.0008 (8)	0.0011 (9)	−0.0069 (9)
C6	0.0434 (10)	0.0346 (9)	0.0501 (11)	0.0033 (7)	−0.0005 (9)	0.0002 (8)
C7	0.0603 (12)	0.0302 (9)	0.0573 (13)	−0.0074 (8)	0.0015 (11)	−0.0072 (9)
C8	0.0477 (10)	0.0302 (8)	0.0455 (10)	−0.0024 (7)	−0.0014 (9)	−0.0011 (8)
C9	0.0537 (11)	0.0453 (11)	0.0551 (12)	−0.0007 (10)	−0.0151 (11)	0.0084 (10)
C10	0.0439 (9)	0.0360 (8)	0.0371 (9)	−0.0064 (7)	−0.0058 (8)	0.0027 (7)
C11	0.0409 (9)	0.0321 (8)	0.0364 (9)	−0.0035 (7)	−0.0031 (7)	−0.0007 (7)
C12	0.0476 (10)	0.0525 (12)	0.0369 (10)	−0.0055 (9)	−0.0060 (9)	−0.0045 (9)
C13	0.0703 (15)	0.0514 (13)	0.0546 (14)	−0.0030 (11)	−0.0120 (12)	−0.0122 (11)
C14	0.099 (2)	0.080 (2)	0.070 (2)	0.0254 (18)	−0.0267 (18)	−0.0332 (18)
C15	0.083 (2)	0.133 (4)	0.0544 (17)	0.037 (2)	−0.0180 (16)	−0.039 (2)
C16	0.0714 (19)	0.161 (4)	0.0392 (13)	0.000 (2)	0.0035 (13)	−0.0025 (19)
C17	0.0645 (15)	0.0838 (19)	0.0401 (12)	−0.0096 (13)	−0.0040 (11)	0.0066 (12)
C18	0.0481 (10)	0.0426 (10)	0.0491 (11)	−0.0088 (8)	0.0016 (10)	−0.0030 (9)
C19	0.0414 (9)	0.0357 (9)	0.0397 (10)	0.0009 (7)	−0.0033 (8)	0.0013 (8)
C20	0.089 (2)	0.0620 (16)	0.079 (2)	−0.0208 (14)	−0.0223 (17)	0.0373 (16)
C21	0.087 (2)	0.0523 (14)	0.091 (2)	0.0249 (14)	−0.002 (2)	0.0125 (14)
N1	0.0629 (11)	0.0336 (8)	0.0543 (10)	0.0054 (8)	−0.0047 (9)	0.0061 (8)
N2	0.0552 (10)	0.0433 (10)	0.0532 (11)	−0.0086 (7)	0.0016 (9)	−0.0114 (8)
N3	0.0382 (9)	0.0719 (13)	0.0573 (12)	−0.0055 (8)	−0.0024 (9)	−0.0161 (11)
O1	0.0878 (13)	0.0495 (10)	0.0729 (12)	−0.0332 (9)	0.0181 (11)	−0.0134 (9)
O2	0.0571 (9)	0.0571 (9)	0.0460 (9)	−0.0035 (7)	−0.0132 (8)	0.0043 (7)
O3	0.0568 (8)	0.0361 (7)	0.0472 (8)	−0.0059 (6)	−0.0068 (7)	0.0092 (6)
O4	0.0599 (11)	0.1159 (19)	0.0916 (18)	−0.0154 (12)	0.0277 (12)	−0.0243 (16)
O5	0.0721 (12)	0.0647 (11)	0.0806 (14)	0.0173 (9)	0.0032 (11)	−0.0056 (11)
F	0.131 (2)	0.222 (4)	0.0689 (15)	0.078 (2)	−0.0131 (14)	−0.0631 (18)

### Geometric parameters (Å, º)

**Table:**

C1—C2	1.389 (4)	C12—C13	1.400 (4)
C1—C6	1.396 (3)	C13—C14	1.374 (4)
C1—H1	0.9300	C13—H13	0.9300
C2—C3	1.375 (4)	C14—C15	1.365 (7)
C2—H2	0.9300	C14—H14	0.9300
C3—C4	1.407 (3)	C15—F	1.363 (4)
C3—H3	0.9300	C15—C16	1.372 (7)
C4—C5	1.374 (3)	C16—C17	1.394 (5)
C4—H4	0.9300	C16—H16	0.9300
C5—C6	1.394 (3)	C17—H17	0.9300
C5—N2	1.414 (3)	C18—N3	1.507 (3)
C6—C8	1.521 (3)	C18—H18A	0.9700
C7—O1	1.226 (3)	C18—H18B	0.9700
C7—N2	1.343 (3)	C19—O2	1.196 (3)
C7—C8	1.554 (3)	C19—O3	1.323 (2)
C8—N1	1.460 (3)	C20—O3	1.447 (3)
C8—C11	1.590 (3)	C20—H20A	0.9600
C9—N1	1.476 (3)	C20—H20B	0.9600
C9—C10	1.545 (3)	C20—H20C	0.9600
C9—H9A	0.9700	C21—N1	1.467 (3)
C9—H9B	0.9700	C21—H21A	0.9600
C10—C12	1.511 (3)	C21—H21B	0.9600
C10—C11	1.573 (3)	C21—H21C	0.9600
C10—H10	0.9800	N2—H2A	0.8600
C11—C18	1.529 (3)	N3—O4	1.207 (3)
C11—C19	1.530 (3)	N3—O5	1.209 (3)
C12—C17	1.392 (4)		
			
C2—C1—C6	119.0 (2)	C13—C12—C10	118.7 (2)
C2—C1—H1	120.5	C14—C13—C12	121.7 (3)
C6—C1—H1	120.5	C14—C13—H13	119.1
C3—C2—C1	121.2 (2)	C12—C13—H13	119.1
C3—C2—H2	119.4	C15—C14—C13	118.1 (3)
C1—C2—H2	119.4	C15—C14—H14	120.9
C2—C3—C4	120.9 (2)	C13—C14—H14	120.9
C2—C3—H3	119.5	F—C15—C14	119.0 (4)
C4—C3—H3	119.5	F—C15—C16	118.0 (4)
C5—C4—C3	116.9 (2)	C14—C15—C16	123.0 (3)
C5—C4—H4	121.6	C15—C16—C17	118.4 (4)
C3—C4—H4	121.6	C15—C16—H16	120.8
C4—C5—C6	123.4 (2)	C17—C16—H16	120.8
C4—C5—N2	127.0 (2)	C12—C17—C16	120.5 (4)
C6—C5—N2	109.5 (2)	C12—C17—H17	119.7
C5—C6—C1	118.5 (2)	C16—C17—H17	119.7
C5—C6—C8	108.73 (18)	N3—C18—C11	114.69 (17)
C1—C6—C8	132.8 (2)	N3—C18—H18A	108.6
O1—C7—N2	126.4 (2)	C11—C18—H18A	108.6
O1—C7—C8	124.8 (2)	N3—C18—H18B	108.6
N2—C7—C8	108.78 (19)	C11—C18—H18B	108.6
N1—C8—C6	119.80 (19)	H18A—C18—H18B	107.6
N1—C8—C7	109.50 (17)	O2—C19—O3	125.5 (2)
C6—C8—C7	101.10 (18)	O2—C19—C11	121.53 (18)
N1—C8—C11	100.84 (17)	O3—C19—C11	112.90 (17)
C6—C8—C11	112.69 (16)	O3—C20—H20A	109.5
C7—C8—C11	113.42 (18)	O3—C20—H20B	109.5
N1—C9—C10	106.07 (17)	H20A—C20—H20B	109.5
N1—C9—H9A	110.5	O3—C20—H20C	109.5
C10—C9—H9A	110.5	H20A—C20—H20C	109.5
N1—C9—H9B	110.5	H20B—C20—H20C	109.5
C10—C9—H9B	110.5	N1—C21—H21A	109.5
H9A—C9—H9B	108.7	N1—C21—H21B	109.5
C12—C10—C9	115.6 (2)	H21A—C21—H21B	109.5
C12—C10—C11	115.70 (16)	N1—C21—H21C	109.5
C9—C10—C11	104.48 (16)	H21A—C21—H21C	109.5
C12—C10—H10	106.8	H21B—C21—H21C	109.5
C9—C10—H10	106.8	C8—N1—C21	114.9 (2)
C11—C10—H10	106.8	C8—N1—C9	107.58 (16)
C18—C11—C19	109.31 (17)	C21—N1—C9	113.0 (2)
C18—C11—C10	113.27 (17)	C7—N2—C5	111.58 (19)
C19—C11—C10	116.74 (15)	C7—N2—H2A	124.2
C18—C11—C8	109.60 (16)	C5—N2—H2A	124.2
C19—C11—C8	107.18 (16)	O4—N3—O5	124.3 (3)
C10—C11—C8	100.04 (15)	O4—N3—C18	116.3 (2)
C17—C12—C13	118.1 (3)	O5—N3—C18	119.3 (2)
C17—C12—C10	123.2 (2)	C19—O3—C20	115.7 (2)
			
C6—C1—C2—C3	1.1 (4)	C9—C10—C12—C17	−34.3 (3)
C1—C2—C3—C4	−1.6 (4)	C11—C10—C12—C17	88.4 (3)
C2—C3—C4—C5	0.4 (4)	C9—C10—C12—C13	145.6 (2)
C3—C4—C5—C6	1.2 (4)	C11—C10—C12—C13	−91.8 (2)
C3—C4—C5—N2	−178.1 (2)	C17—C12—C13—C14	−3.0 (4)
C4—C5—C6—C1	−1.7 (3)	C10—C12—C13—C14	177.2 (2)
N2—C5—C6—C1	177.79 (19)	C12—C13—C14—C15	1.0 (5)
C4—C5—C6—C8	177.1 (2)	C13—C14—C15—F	−179.0 (3)
N2—C5—C6—C8	−3.4 (2)	C13—C14—C15—C16	0.8 (5)
C2—C1—C6—C5	0.5 (3)	F—C15—C16—C17	179.3 (3)
C2—C1—C6—C8	−177.9 (2)	C14—C15—C16—C17	−0.6 (5)
C5—C6—C8—N1	125.3 (2)	C13—C12—C17—C16	3.2 (4)
C1—C6—C8—N1	−56.2 (3)	C10—C12—C17—C16	−177.0 (3)
C5—C6—C8—C7	5.0 (2)	C15—C16—C17—C12	−1.5 (5)
C1—C6—C8—C7	−176.4 (2)	C19—C11—C18—N3	−57.6 (2)
C5—C6—C8—C11	−116.37 (19)	C10—C11—C18—N3	74.4 (2)
C1—C6—C8—C11	62.2 (3)	C8—C11—C18—N3	−174.82 (19)
O1—C7—C8—N1	45.3 (3)	C18—C11—C19—O2	−49.3 (3)
N2—C7—C8—N1	−132.4 (2)	C10—C11—C19—O2	−179.5 (2)
O1—C7—C8—C6	172.7 (2)	C8—C11—C19—O2	69.4 (3)
N2—C7—C8—C6	−5.1 (2)	C18—C11—C19—O3	133.43 (19)
O1—C7—C8—C11	−66.4 (3)	C10—C11—C19—O3	3.2 (2)
N2—C7—C8—C11	115.8 (2)	C8—C11—C19—O3	−107.87 (19)
N1—C9—C10—C12	133.5 (2)	C6—C8—N1—C21	−45.7 (3)
N1—C9—C10—C11	5.2 (2)	C7—C8—N1—C21	70.3 (3)
C12—C10—C11—C18	−41.3 (2)	C11—C8—N1—C21	−169.9 (2)
C9—C10—C11—C18	87.1 (2)	C6—C8—N1—C9	81.1 (2)
C12—C10—C11—C19	87.0 (2)	C7—C8—N1—C9	−162.96 (19)
C9—C10—C11—C19	−144.63 (19)	C11—C8—N1—C9	−43.2 (2)
C12—C10—C11—C8	−157.81 (18)	C10—C9—N1—C8	24.4 (2)
C9—C10—C11—C8	−29.5 (2)	C10—C9—N1—C21	152.3 (2)
N1—C8—C11—C18	−75.4 (2)	O1—C7—N2—C5	−174.3 (3)
C6—C8—C11—C18	155.62 (19)	C8—C7—N2—C5	3.4 (3)
C7—C8—C11—C18	41.5 (2)	C4—C5—N2—C7	179.4 (2)
N1—C8—C11—C19	166.04 (16)	C6—C5—N2—C7	0.0 (3)
C6—C8—C11—C19	37.1 (2)	C11—C18—N3—O4	−153.5 (2)
C7—C8—C11—C19	−77.0 (2)	C11—C18—N3—O5	30.2 (3)
N1—C8—C11—C10	43.83 (18)	O2—C19—O3—C20	−0.8 (4)
C6—C8—C11—C10	−85.1 (2)	C11—C19—O3—C20	176.3 (2)
C7—C8—C11—C10	160.79 (18)		

### Hydrogen-bond geometry (Å, º)

**Table:**

*D*—H···*A*	*D*—H	H···*A*	*D*···*A*	*D*—H···*A*
N2—H2*A*···N1^i^	0.86	2.34	3.127 (2)	152
C4—H4···O1^i^	0.93	2.42	3.223 (2)	145
C9—H9*B*···O1^ii^	0.97	2.49	3.314 (2)	142
C9—H9*A*···O4^iii^	0.97	2.51	3.443 (3)	160

Symmetry codes: (i) −*x*, −*y*, *z*−1/2; (ii) −*x*, −*y*, *z*+1/2; (iii) *x*+1, *y*, *z*.
